# Introduction of Two Prolines and Removal of the Polybasic Cleavage Site Lead to Higher Efficacy of a Recombinant Spike-Based SARS-CoV-2 Vaccine in the Mouse Model

**DOI:** 10.1128/mBio.02648-20

**Published:** 2021-03-02

**Authors:** Fatima Amanat, Shirin Strohmeier, Raveen Rathnasinghe, Michael Schotsaert, Lynda Coughlan, Adolfo García-Sastre, Florian Krammer

**Affiliations:** a Department of Microbiology, Icahn School of Medicine at Mount Sinai, New York, New York, USA; b Graduate School of Biomedical Sciences, Icahn School of Medicine at Mount Sinai, New York, New York, USA; c Global Health and Emerging Pathogens Institute, Icahn School of Medicine at Mount Sinai, New York, New York, USA; d Department of Medicine, Division of Infectious Diseases, Icahn School of Medicine at Mount Sinai, New York, New York, USA; e The Tisch Cancer Institute, Icahn School of Medicine at Mount Sinai, New York, New York, USA; Columbia University College of Physicians & Surgeons

**Keywords:** COVID-19, SARS-CoV-2, spike, vaccine

## Abstract

The spike protein of severe acute respiratory syndrome coronavirus 2 (SARS-CoV-2) has been identified as the prime target for vaccine development. The spike protein mediates both binding to host cells and membrane fusion and is also so far the only known viral target of neutralizing antibodies. Coronavirus spike proteins are large trimers that are relatively unstable, a feature that might be enhanced by the presence of a polybasic cleavage site in SARS-CoV-2 spike. Exchange of K986 and V987 for prolines has been shown to stabilize the trimers of SARS-CoV-1 and the Middle East respiratory syndrome coronavirus spike proteins. Here, we test multiple versions of a soluble spike protein for their immunogenicity and protective effect against SARS-CoV-2 challenge in a mouse model that transiently expresses human angiotensin-converting enzyme 2 via adenovirus transduction. Variants tested include spike proteins with a deleted polybasic cleavage site, proline mutations, or a combination thereof, besides the wild-type protein. While all versions of the protein were able to induce neutralizing antibodies, only the antigen with both a deleted cleavage site and the K986P and V987P (PP) mutations completely protected from challenge in this mouse model.

## Abstract (precis)

A vaccine for SARS-CoV-2 is urgently needed. A better understanding of antigen design and attributes that vaccine candidates need to have to induce protective immunity is of high importance. The data presented here validate the choice of antigens that contain the PP mutations and suggest that deletion of the polybasic cleavage site may lead to a further-optimized design.

## INTRODUCTION

Severe acute respiratory syndrome coronavirus 2 (SARS-CoV-2) emerged in late 2019 in China and has since caused the coronavirus disease 2019 (COVID-19) pandemic (1–3). Vaccines are an urgently needed countermeasure to the virus. Vaccine candidates have been moved at unprecedented speed through the pipeline, with the first phase III trials already taking place in the summer of 2020, only half a year after discovery of the virus sequence. From studies on SARS-CoV-1 and Middle East respiratory syndrome CoV (MERS-CoV), it was clear that the spike protein of the virus is the best target for vaccine development (4–6). Most CoVs have only one large surface glycoprotein (a minority also have a hemagglutinin [HA] esterase) that is used by the virus to attach to the host cell and trigger the fusion of viral and cellular membranes. The spike protein of SARS-CoV-2, like the one of SARS-CoV-1, binds to human angiotensin-converting enzyme 2 (hACE2) (7–9). In order to be able to trigger fusion, the spike protein has to be cleaved into the S1 and S2 subunits (10–12). Additionally, a site in S2 (S2′) that has to be cleaved to activate the fusion machinery has been reported as well (13). While the spike of SARS-CoV-1 contains a single basic amino acid at the cleavage site between S1 and S2, SARS-CoV-2 has a polybasic motif that can be activated by furin-like proteases (10–12), analogously to the HA of highly pathogenic H5 and H7 avian influenza viruses. In addition, it has been reported that the activated spike protein of CoVs is relatively unstable and that multiple conformations might exist, of which not all may present neutralizing epitopes to the immune system. For SARS-CoV-1- and MERS-CoV-stabilizing mutations—a pair of prolines replacing K986 and V987 in S2—have been described (14), and a beneficial effect on stability has also been shown for SARS-CoV-2 (9). Here, we set out to investigate if including these stabilizing mutations, removing the cleavage site between S1 and S2, or combining the two strategies to stabilize the spike would increase its immunogenicity and protective effect in a mouse model that transiently expresses hACE2 via adenovirus (AdV) transduction (15). This information is important since it can help to optimize vaccine candidates, especially improved versions of vaccines that might be licensed at a later point in time.

## RESULTS

### Construct design and recombinant protein expression.

**FIG 1:**
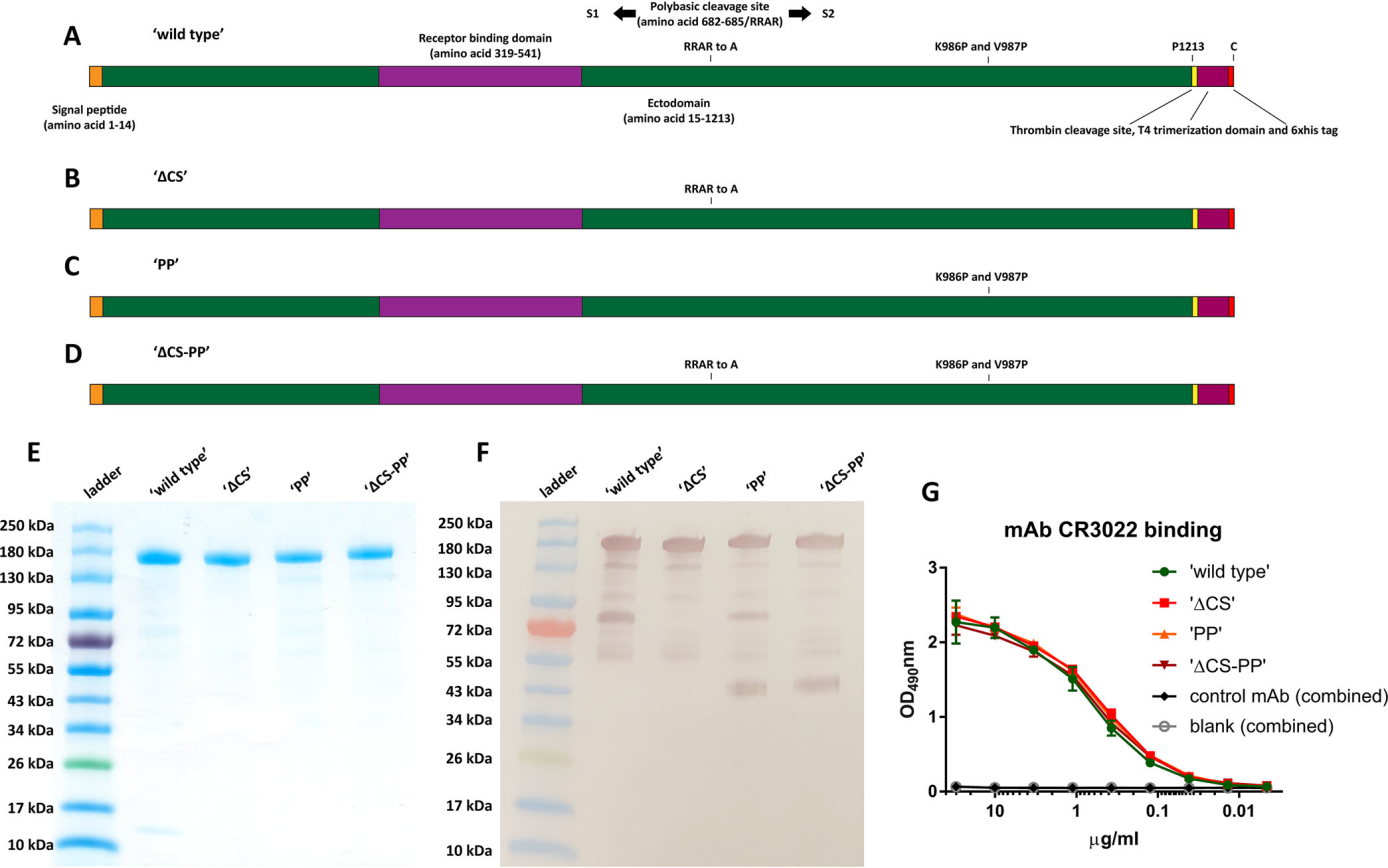
Spike construct design and protein characterization. (A to D) Illustration of the wild-type, ΔCS, PP, and ΔCS-PP constructs used in this study. (E) Four antigens on an SDS-PAGE gel stained with Coomassie blue. (F) The same proteins on a Western blot developed with an antibody to the C-terminal hexahistidine tag. While all four proteins are detected as clean, single bands on the SDS-PAGE gel, the Western blot reveals a small fraction of degradation products at approximately 80 kDa for the wild type and PP variants and of approximately 40 kDa for the PP and ΔCS-PP constructs. (G) Binding of MAb CR3022 to the constructs in an ELISA. Data for the negative-control MAb and the blank were combined for the different substrates.

The sequence based on the S gene of SARS-CoV-2 strain Wuhan-1 was initially codon optimized for mammalian cell expression. The wild-type signal peptide and ectodomain (amino acids 1 to 1213) were fused to a T4 foldon trimerization domain followed by a hexahistidine tag to facilitate purification. This construct was termed wild type (WT). Additional constructs were generated, including one in which the polybasic cleavage site (RRAR) was replaced by a single alanine (termed ΔCS), one in which K986 and V987 in the S2 subunit were mutated to prolines (PP), and one in which both modifications were combined (ΔCS-PP) (Fig. 1A to C). The proteins were then expressed in a baculovirus expression system and purified. At first inspection by sodium dodecyl sulfate polyacrylamide gel electrophoresis (SDS-PAGE) and Coomassie blue staining, all four constructs appeared similar, with a major clean band at approximately 180 kDa (Fig. 1E). When Western blotting was performed, additional bands were detected in the lanes with the WT, PP, and ΔCS-PP constructs, suggesting cleavage of a fraction of the protein. However, the patterns of the bands were different for the three constructs. For the WT, the most prominent detected smaller band ran at 80 kDa, was visualized with an antibody recognizing the C-terminal hexahistidine tag, and likely represents S2 (Fig. 1F). The two constructs containing the PP mutations also produced an additional band at approximately 40 kDa (Fig. 1E), potentially representing a fragment downstream of S2′. While in general, these bands were invisible by SDS-PAGE and therefore likely represent only a tiny fraction of the purified spike protein, they might indicate vulnerability to proteolytic digest of the antigen *in vivo*. All constructs were also recognized in a similar manner by monoclonal antibody (MAb) CR3022 (16, 17), an antibody that binds to the receptor binding domain (RBD) (Fig. 1F).

### All versions of the recombinant spike protein induce robust immune responses in mice.

**FIG 2:**
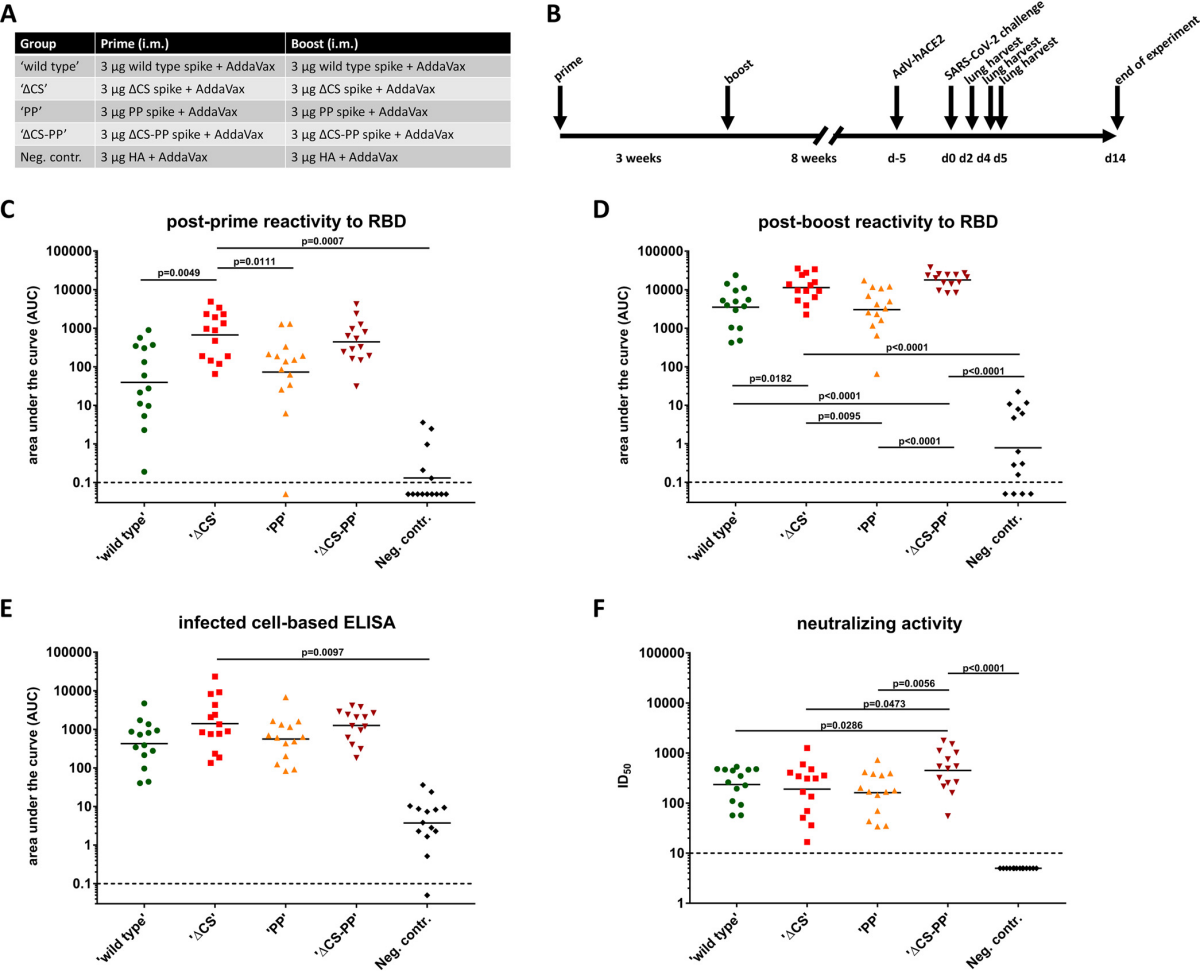
Immunogenicity of different spike variants in the mouse model. (A) Vaccination regimen used for the five groups of mice. (B) Timeline. d-5 and d5, day -5 and day 5. (C and D) Animals were bled 3 weeks after the priming (C) and 4 weeks after the booster (D), and levels of antibody to a mammalian-cell-expressed RBD were measured. Postboost sera were also tested in cell-based ELISAs on cells infected with authentic SARS-CoV-2. Finally, postboost sera were tested in a microneutralization assay against SARS-CoV-2.

To test the immunogenicity of the four spike constructs, all proteins were used in a simple prime-boost study in mice (Fig. 2A). Animals were injected intramuscularly (i.m.) with 3 μg of spike protein adjuvanted with AddaVax (a generic version of the oil-in-water adjuvant MF59) twice in a 3-week interval. A control group received an irrelevant immunogen, recombinant influenza virus hemagglutinin (HA), also expressed in insect cells, with AddaVax. Mice were bled 3 weeks after the priming and 4 weeks after the boost to assess the immune response that they mounted to the vaccine (Fig. 2B). To determine antibody levels to the RBD, we performed enzyme-linked immunosorbent assays (ELISAs) against the recombinant, mammalian cell-expressed RBD (18, 19). All animals (except the negative control animals) made anti-RBD responses after the priming, but they were higher in the ΔCS and ΔCS-PP groups than in the WT or PP group (Fig. 2C). The booster dose increased antibodies to the RBD significantly, but the same pattern persisted (Fig. 2D). Interestingly, the ΔCS-PP group showed very homogenous responses compared to those of the other groups, in which there was more spread between the animals. In addition, we performed cell-based ELISAs with Vero cells infected with SARS-CoV-2 as the target. While all groups showed good reactivity, similar patterns emerged in which ΔCS and ΔCS-PP groups showed higher reactivity than WT and PP groups (Fig. 2E). Finally, we performed microneutralization assays with authentic SARS-CoV-2 (20). Here, the WT, PP, and ΔCS groups showed similar levels of neutralization, while the ΔCS-PP group of animals had higher serum neutralization titers (Fig. 2F).

### Vaccination with recombinant S protein variants protects mice from challenge with SARS-CoV-2.

**FIG 3:**
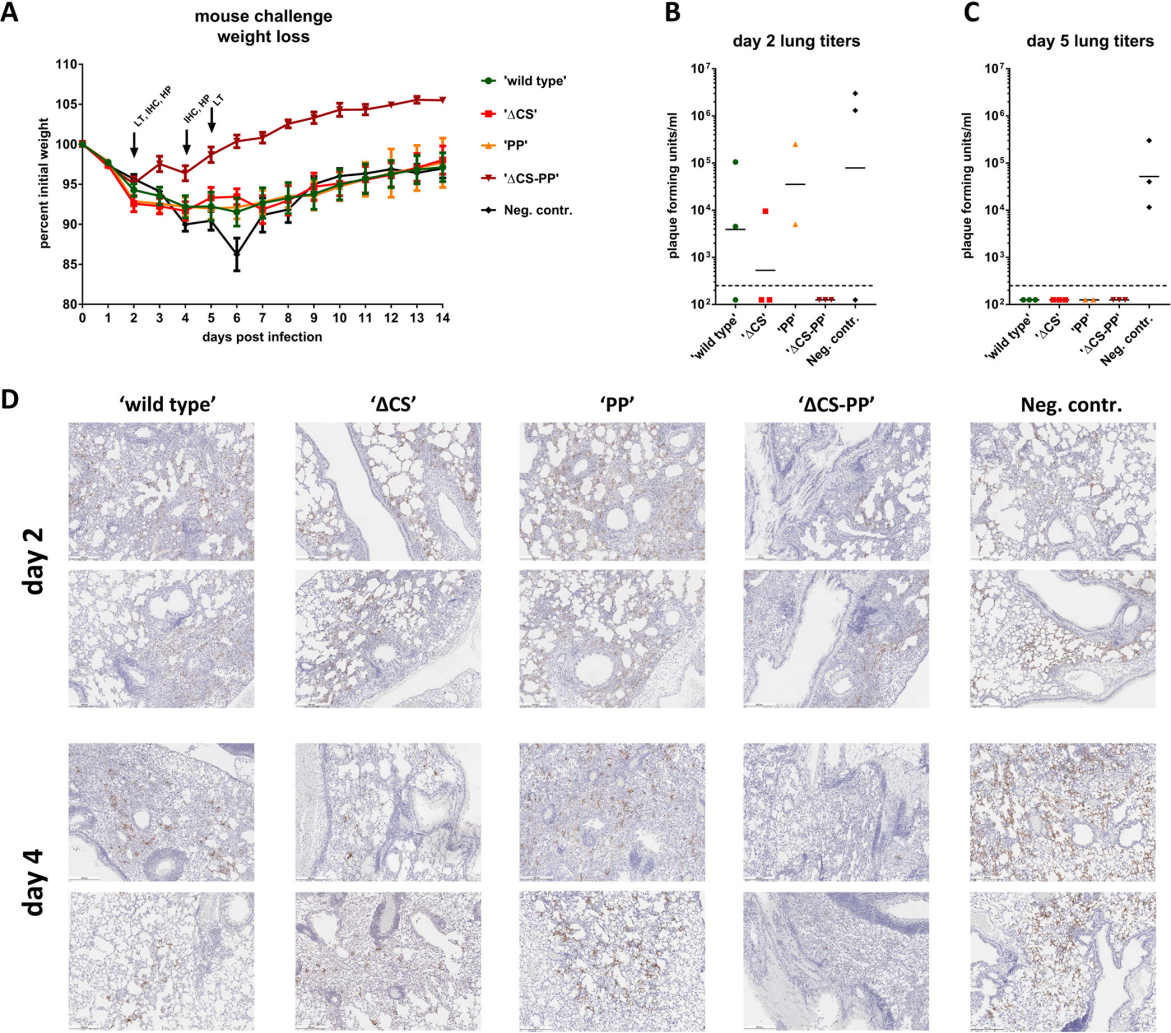
Challenge of mice with SARS-CoV-2. Animals sensitized by transient expression of hACE2 via adenovirus transduction were challenged with 10^5^ PFU of SARS-CoV-2, and weight loss was monitored over a period of 14 days (A). (B and C) Day 2 and day 5 lung titers, respectively. (D) Lung immunohistochemistry staining for SARS-CoV-2 nucleoprotein on days 2 and 4 postchallenge. Representative images from two animals each are shown at a 5-fold magnification. Scale bar = 500 μm.

In order to perform challenge studies, mice were sensitized to infection with SARS-CoV-2 by intranasal (i.n.) transduction with an adenovirus expressing hACE2 (AdV-hACE2), using a treatment regimen described previously (Fig. 2A) (15, 21, 22). They were then challenged with 10^5^ plaque forming units (PFU) of SARS-CoV-2 and monitored for weight loss and mortality for 14 days. Additional animals were euthanized on day 2 and day 4 to harvest lungs for histopathological assessment and immunohistochemistry (IHC) and on day 2 and day 5 to measure virus titers in the lung. After challenge, all groups lost weight, trending with the negative-control group (irrelevant HA protein vaccination), except for the ΔCS-PP group, which displayed minimal weight loss (Fig. 3A). Only on days 4 to 6, the WT, PP, and ΔCS groups showed a trend toward less weight loss than the control group. However, all animals recovered, and by day 14, no mortality was observed. Lung titers on day 2 suggested low virus replication in the WT, PP, and ΔCS groups, with some animals having no detectable virus and no presence of replication-competent virus in the ΔCS-PP animals (Fig. 3B). Two of the control animals showed high virus replication, while virus could not be recovered from the third animal. No virus could be detected in any of the vaccinated groups on day 5, while all three controls still had detectable virus in the range of 10^4^ to 10^5^ PFU (Fig. 3C).

### Lung immunohistochemistry and pathology.

**FIG 4:**
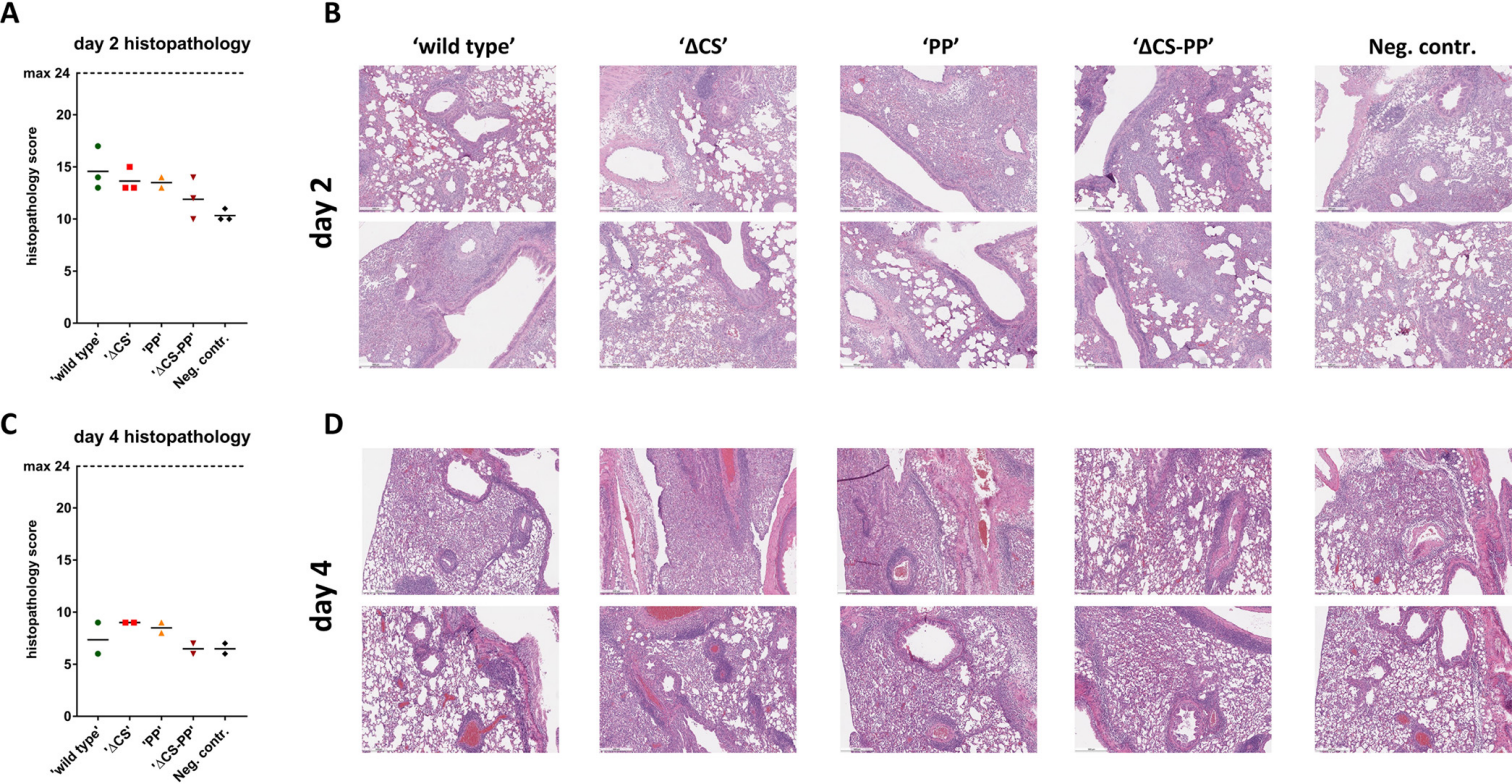
Lung pathology. (A) Histopathological composite scores for animals on day 2 postinfection; (B) representative H&E-stained tissue images from 2 animals per group; (C and D) the same tissues but for day 4 postchallenge. Scale bar = 500 μm.

Lungs were harvested on days 2 and 4 postchallenge. Samples from both days were used for immunohistochemistry to detect viral nucleoprotein antigen. Viral antigen was detectable in all groups on day 2 as well as day 4 postinfection (Fig. 3D). However, the ΔCS-PP group showed very few positive cells, especially on day 4, while antigen was more widespread in all other groups. These results correlate well with the viral lung titers reported above. The samples were also stained with hematoxylin and eosin (H&E) and scored for lung pathology by a qualified veterinary pathologist using a composite score with a maximum value of 24 (Fig. 4A and C). At day 2 postinfection with SARS-CoV-2, all mice were determined to exhibit histopathological lesions typical of interstitial pneumonia, with more severe alveolar inflammation in the WT group. Alveolar congestion and edema were also more pronounced in S-vaccinated groups than in groups vaccinated with the irrelevant control HA immunogen. At this time point, the overall pathology score was lowest for the irrelevant HA control group, followed by ΔCS-PP<PP<ΔCS<wild type (Fig. 4A). On day 4, all groups showed mild-to-moderate pathology scores, reduced in severity compared with those on day 2. Observations included perivascular, bronchial, and alveolar inflammation, as well as mild-to-moderate congestion or edema. Scores were slightly higher in vaccinated than in control animals, which may reflect the infiltration of CoV-2 antigen-specific immune cells into the lung, which are absent in the irrelevant HA-immunized control mice (Fig. 4C and D).

## DISCUSSION

The spike protein of SARS-CoV-2 was selected early on as a target for vaccine development, based on experience with SARS-CoV-1 and MERS-CoV (6). The coronavirus spike protein is known to be relatively labile, and in addition to this inherent property, the SARS-CoV-2 spike protein contains a polybasic cleavage site between S1 and S2. Work on SARS-CoV-1 and MERS-CoV had shown that introducing two prolines in positions 986 and 987 (SARS-CoV-2 numbering) improves stability and expression (14). In addition, removal of polybasic cleavage sites has been shown to stabilize hemagglutinin (HA) proteins of highly pathogenic influenza viruses. In this study, we tested different versions of the protein either lacking the polybasic cleavage site or including the stabilizing PP mutations or both. While vaccination with all constructs induced neutralizing antibodies and led to control of virus replication in the lung, we observed notable differences. Removing the polybasic cleavage side did increase the humoral immune response in ELISAs. Since we did not observe cleavage of the majority of the protein when it was purified, even with the polybasic cleavage site present (although some cleavage could be observed), we speculate that removal of the site might make the protein more stable *in vivo* postvaccination. Longer stability may lead to stronger and potentially more uniform immune responses. The combination of deleting the polybasic cleavage site plus introducing the PP mutations performed best, also in terms of protection of mice from weight loss. It is important to note that all versions of the protein tested had a third stabilizing element present, which is a trimerization domain. This trimerization domain might have also increased stability and immunogenicity.

Current leading vaccine candidates in clinical trials and licensed/authorized vaccines include virus-vectored and mRNA vaccines as well as inactivated vaccines and recombinant protein vaccines (23). The ChAdOx-based vaccine candidate developed by AstraZeneca, as well as the CanSino- and Gamaleya-vectored candidates, use a wild-type version of the spike protein (24–26). The same is of course true for the inactivated vaccines produced by Sinovac and Sinopharm (27, 28). Moderna’s and Pfizer’s mRNA vaccines are based on a spike construct that includes the PP mutations but features a wild-type cleavage site (29, 30). It is currently unclear if addition of the modifications shown here to enhance the immunogenicity of recombinant protein spike antigens would also enhance the immunogenicity of these constructs. However, it might be worth testing if these vaccine candidates can be improved by our strategy as well. Of note, one study in nonhuman primates with adenovirus 26-vectored vaccine candidates (from J&J) expressing different versions of the spike protein also showed that the ΔCS-PP candidate (although including the transmembrane domain) performed best, and this candidate was moved forward into clinical trials and is expected to be authorized/licensed soon (31). Similarly, Novavax is using a recombinant spike construct that features ΔCS-PP and, when adjuvanted, induced high neutralization titers in humans in a phase I clinical trial (32).

While vaccination with all constructs led to various degrees of control of virus replication, histopathology scores, especially on day 2 after challenge, were above those of the negative-control animals. The histopathology scores higher than those of the negative controls are likely due to an antigen-specific immune response and not due to the adenovirus transduction, which in general leads to only transient and mild inflammation (and would also be present in the control group) (15, 21). This is also evidenced by significantly reduced weight loss in the ΔCS-PP group as well as complete control of virus replication, despite increased lung histopathology scores. However, future studies with recombinant protein vaccines that are routed for clinical testing will need to assess this increase in lung pathology in more detail. Other caveats that need to be discussed are the variability introduced by the adenovirus transduction step and the many cell types that are transduced by human adenovirus 5 (HAdV-C5) in mice, including cell types that do not express ACE2 in humans (33–35). The first point may explain why some animals did not have viral titers on day 2, while the second point may explain why, despite high neutralization titers, protection was suboptimal in several of the groups.

Recombinant protein vaccines including the spike ectodomain (36, 37) and membrane-extracted spike (38), as well as S1 (39) and the RBD (40), have been tested for SARS-CoV-1, and several studies show good efficacy against challenge in animal models. It is, therefore, not surprising that similar constructs for SARS-CoV-2 also provided protection. While our goal was not vaccine development but studying the effect of stabilizing elements on the immunogenicity of the spike protein, Sanofi Pasteur has announced the development of a recombinant protein-based SARS-CoV-2 vaccine, and several additional recombinant protein vaccine candidates are being developed with Novavax' candidate expected to be licensed/authorized soon. Our data show that this approach might be effective.

## MATERIALS AND METHODS

### Cells and viruses.

Vero.E6 cells (ATCC CRL‐1586, clone E6) were maintained in culture using Dulbecco's modified Eagle medium (Gibco), which was supplemented with an antibiotic-antimycotic mixture (100 U/ml penicillin–100 μg/ml streptomycin–0.25 μg/ml amphotericin B) (Gibco; 15240062) and 10% fetal bovine serum (FBS; Corning). SARS-CoV-2 (isolate USA‐WA1/2020; BEI Resources, catalog no. NR‐52281) was grown in Vero.E6 cells as previously described and was used for the *in vivo* challenge (20). A viral seed stock for a nonreplicating human adenovirus type 5 (HAdV-C5) vector expressing the human ACE2 receptor was obtained from the Iowa Viral Vector Core Facility. High-titer AdV-hACE2 stocks were amplified in TRex-293 cells and purified by CsCl ultracentrifugation, and infectious titers were determined by 50% tissue culture infectious dose (TCID_50_) analysis, with adjustment for PFU titers using the Kärber statistical method, as described previously (41).

### Recombinant proteins.

All recombinant proteins were expressed and purified using the baculovirus expression system, as previously described (18, 42, 43). Different versions of the spike protein of SARS-CoV-2 (GenBank accession no. MN908947.3 for the original sequence, accession no. MT380725 for the codon-optimized ΔCS-PP construct) were expressed to assess immunogenicity. PP indicates that two stabilizing prolines were induced at K986 and K987. ΔCS indicates that the cleavage site of the spike protein was removed by deletion of the arginine residues (RRAR to just A). The HA was also produced in the baculovirus expression system, as with the spike variants.

### SDS-PAGE and Western blotting.

One microgram of each respective protein was mixed at a 1:1 ratio with 2× Laemmli buffer (Bio-Rad), which was supplemented with 2% β-mercaptoethanol (Fisher Scientific). The samples were heated at 90°C for 10 min and loaded onto a 4 to 20% precast polyacrylamide gel (Bio-Rad). The gel was stained with SimplyBlue SafeStain (Invitrogen) for 1 h and then destained with water for a few hours. For Western blotting, the same process as mentioned above was used. After the gel was run, the gel was transferred onto a nitrocellulose membrane, as described previously (42). The membrane was blocked with phosphate-buffered saline (PBS; Gibco) containing 3% nonfat milk (AmericanBio; catalog no. AB10109‐01000) for an hour at room temperature on an orbital shaker. Next, primary antibody was prepared in PBS containing 1% nonfat milk using antihexahistidine antibody (TaKaRa Bio; catalog no. 631212) at a dilution of 1:3,000. The membrane was stained with primary antibody solution for 1 h at room temperature. The membrane was washed three times with PBS containing 0.1% Tween 20 (PBS-T; Fisher Scientific). The secondary solution was prepared with 1% nonfat milk in PBS-T using anti-mouse IgG (whole molecule)–alkaline phosphatase (AP) antibody produced in goat (Sigma-Aldrich) at a dilution of 1:3,000. The membrane was developed using an AP conjugate substrate kit (catalog no. 1706432; Bio-Rad).

### ELISA.

Ninety-six-well plates (Immulon 4 HBX; ThermoFisher Scientific) were coated with the recombinant RBD at a concentration of 2 μg/ml, with 50 μl/well overnight. The RBD protein was produced in 293F cells and purified using Ni-nitrilotriacetic acid (Ni-NTA) resin, and this procedure has been described in detail previously (19). The next morning, coating solution was removed and plates were blocked with 100 μl of 3% nonfat milk (AmericanBio; catalog no. AB10109‐01000) prepared in PBS-T for 1 h at room temperature (RT). Serum samples from vaccinated mice were tested in an ELISA, starting at a dilution of 1:50, and subsequent 3-fold dilutions were performed. Serum samples were prepared in PBS-T containing 1% nonfat milk, and the plates were incubated with the serum samples for 2 h at RT. Next, plates were washed with 200 μl of PBS-T three times. Anti-mouse IgG conjugated to horseradish peroxidase (Rockland; catalog no. 610‐4302) was used at a concentration of 1:3,000 in PBS-T with 1% nonfat milk, and 100 μl was added to each well for 1 h at RT. Plates were then washed again with 200 μl of PBS-T and patted dry with a paper towel. Developing solution was prepared in sterile water (WFI; Gibco) using SigmaFast OPD (*o*-phenylenediamine dihydrochloride, catalog no. P9187; Sigma‐Aldrich), and 100 μl was added to each well for a total of 10 min. Next, the reaction was stopped with 50 μl of 3 M hydrochloric acid, and absorbance was measured at 490 nm (OD_490_) using a Synergy 4 (BioTek) plate reader. Data were analyzed using GraphPad Prism 7, and area under the curve (AUC) values were measured and graphed (18). An AUC of 0.05 was assigned to negative values for data analysis purposes. ELISAs with CR3022 against the different versions of recombinant protein were performed in a similar fashion but with an anti-human IgG secondary antibody.

To perform an ELISA on infected cells, Vero.E6 cells were seeded at 20,000 cells per well in a 96-well cell culture plate a day and infected at a multiplicity of infection of 0.1 for 24 h with SARS-CoV-2 (isolate USA‐WA1/2020, catalog no. NR‐52281; BEI Resources). The cells were fixed with 10% formaldehyde (Polysciences) for 24 h, after which the ELISA procedure mentioned above was performed using serum from each vaccinated animal.

### Mouse vaccinations and challenge.

All animal procedures were performed by adhering to the Institutional Animal Care and Use Committee (IACUC) guidelines. Six- to 8-week-old female BALB/c mice (Jackson Laboratories) were immunized intramuscularly with 3 μg of recombinant protein per mouse with an adjuvant, AddaVax (InvivoGen), in a volume of 50 μl. Three weeks later, mice were again immunized, via the intramuscular route, with 3 μg of each respective protein with adjuvant. Mice were bled 3 weeks after the priming regimen and were also bled 4 weeks after the booster regimen. Another 4 weeks later, 2.5 × 10^8^ PFU/mouse of AdV-hACE2 was administered intranasally to each mouse in a final volume of 50 μl sterile PBS. Adhering to institutional guidelines, a mixture containing 0.15 mg/kg of body weight ketamine and 0.03 mg/kg xylazine in water was used as anesthesia for mouse experiments, and intranasal infection was performed under anesthesia.

Five days postadministration of AdV-hACE2, mice were infected with 10^5^ PFU of SARS-CoV-2. On day 2 and day 5, mice were euthanized using humane methods, and the whole lung was dissected from each mouse. Mice were sacrificed for measuring viral titers in the lung as well as to see pathological changes in the lungs. For measuring lung titers, lungs were homogenized using a BeadBlaster 24 (Benchmark) homogenizer, after which the supernatant was clarified by centrifugation at 14,000 × *g* for 10 min. The experimental design was adapted from earlier reported work (15, 44). The remaining mice were weighed daily for 14 days.

### Microneutralization assays.

We used a very detailed protocol that we published earlier for measuring neutralizing antibody in serum samples (18, 20). Briefly, Vero.E6 cells were seeded at a density of 20,000 cells per well in a 96-well cell culture plate. Serum samples were heat inactivated for 1 h at 56°C. Serial dilutions starting at 1:10 were prepared in 1× minimal essential medium (MEM) supplemented with 1% FBS. The remaining steps of the assay were performed in a biosafety level 3 (BSL3) facility. Six hundred TCID_50_s of virus in 80 μl was added to 80 μl of each serum dilution. The serum-virus mixture was incubated at room temperature for 1 h. After 1 h, medium from the cells was removed and 120 μl of the serum-virus mixture was added onto the cells. The cells were incubated for 1 h in a 37°C incubator. After 1 h, all of the serum-virus mixture was removed. One hundred microliters of each corresponding serum dilution was added onto the cells, and 100 μl of 1× MEM was added to the cells as well. The cells were incubated at 37°C for 2 days. After 2 days, cells were fixed with 10% formaldehyde (Polysciences). The next day, cells were stained with an antinucleoprotein antibody (ThermoFisher; catalog no. PA5-81794) according to our published protocol (20). The 50% inhibitory dilution (ID_50_) for each serum was calculated, and the data were graphed. Negative samples were reported as half of the limit of detection (ID_50_ of 5).

### Plaque assays.

Four hundred thousand Vero.E6 cells were plated the day before the plaque assay was performed. All assays using SARS-CoV-2 were performed in the BSL3 facility according to institutional guidelines. To assess viral titers in the lung, plaque assays were performed using lung homogenates. Dilutions of lung homogenates were prepared starting from 10^−1^ to 10^−6^ in 1× MEM supplemented with 2% FBS. Medium was removed from the cells, and each dilution was added to the cells. The cells were incubated in a humidified incubator at 37°C for 1 h. Next, the virus was removed, and cells were overlaid with 2× MEM supplemented with 2% Oxoid agar (final concentration of 0.7%) as well as 4% FBS. The cells were incubated at 37°C for 72 h, after which cells were fixed with 1 ml of 10% formalin (Polysciences) for 24 h to ensure inactivation of the virus. Crystal violet was used to visualize the plaques. The limit of detection was 250.

### Histology and immunohistochemistry.

Mice were subjected to terminal anesthesia and euthanasia, performed by exsanguination of the femoral artery, before lungs were flushed/inflated with 10% formaldehyde by injecting a 19-gauge needle through the trachea on day 4 for immunohistochemistry. Fixed lungs were sent to a commercial entity, Histowiz, for paraffin embedding, tissue analysis, and scoring by an independent veterinary pathologist. Hematoxylin and eosin (H&E) and immunohistochemistry (IHC) staining were performed. IHC staining was performed using an anti-SARS-CoV nucleoprotein antibody (Novus Biologicals; catalog no. NB100-56576). Histology and IHC for day 2 samples were performed on only half of the lung, which was dissected and cut in half from sacrificed mice. The other half of the lung was used for quantification of the virus, as mentioned above.

Scores were assigned by the pathologist based on six parameters: perivascular inflammation, bronchial/bronchiolar epithelial degeneration/necrosis, bronchial/bronchiolar inflammation, intraluminal debris, alveolar inflammation, and congestion/edema. A 5-point scoring system ranging from 0 to 4 was used, with 0 indicating, e.g., no epithelial degeneration/necrosis or inflammation and with 4 indicating severe epithelial degeneration/necrosis and inflammation.

### Statistics.

Statistical analysis was performed in GraphPad Prism using one-way analysis of variance (ANOVA), with results corrected for multiple comparisons.

### Data availability.

Raw data are available from the corresponding author upon reasonable request.

## References

[1] Zhou P, Yang XL, Wang XG, Hu B, Zhang L, Zhang W, Si HR, Zhu Y, Li B, Huang CL, Chen HD, Chen J, Luo Y, Guo H, Jiang RD, Liu MQ, Chen Y, Shen XR, Wang X, Zheng XS, Zhao K, Chen QJ, Deng F, Liu LL, Yan B, Zhan FX, Wang YY, Xiao GF, Shi ZL. 2020. A pneumonia outbreak associated with a new coronavirus of probable bat origin. Nature 588:E6–E6. doi:10.1038/s41586-020-2012-7. 33199918 PMC9744119

[2] Zhu N, Zhang D, Wang W, Li X, Yang B, Song J, Zhao X, Huang B, Shi W, Lu R, Niu P, Zhan F, Ma X, Wang D, Xu W, Wu G, Gao GF, Tan W, Team CNCIaR. 2020. A novel coronavirus from patients with pneumonia in China, 2019. N Engl J Med 382:727–733. doi:10.1056/NEJMoa2001017. 31978945 PMC7092803

[3] Wu F, Zhao S, Yu B, Chen YM, Wang W, Song ZG, Hu Y, Tao ZW, Tian JH, Pei YY, Yuan ML, Zhang YL, Dai FH, Liu Y, Wang QM, Zheng JJ, Xu L, Holmes EC, Zhang YZ. 2020. A new coronavirus associated with human respiratory disease in China. Nature 579:265–269. doi:10.1038/s41586-020-2202-3. 32015508 PMC7094943

[4] Yong CY, Ong HK, Yeap SK, Ho KL, Tan WS. 2019. Recent advances in the vaccine development against Middle East respiratory syndrome-coronavirus. Front Microbiol 10:1781. doi:10.3389/fmicb.2019.01781. 31428074 PMC6688523

[5] Roper RL, Rehm KE. 2009. SARS vaccines: where are we? Expert Rev Vaccines 8:887–898. doi:10.1586/erv.09.43. 19538115 PMC7105754

[6] Amanat F, Krammer F. 2020. SARS-CoV-2 vaccines: status report. Immunity 52:583–589. doi:10.1016/j.immuni.2020.03.007. 32259480 PMC7136867

[7] Letko M, Marzi A, Munster V. 2020. Functional assessment of cell entry and receptor usage for SARS-CoV-2 and other lineage B betacoronaviruses. Nat Microbiol 5:562–569. doi:10.1038/s41564-020-0688-y. 32094589 PMC7095430

[8] Lan J, Ge J, Yu J, Shan S, Zhou H, Fan S, Zhang Q, Shi X, Wang Q, Zhang L, Wang X. 2020. Crystal structure of the 2019-nCoV spike receptor-binding domain bound with the ACE2 receptor. bioRxiv doi:10.1101/2020.02.19.956235. 32225176

[9] Wrapp D, Wang N, Corbett KS, Goldsmith JA, Hsieh CL, Abiona O, Graham BS, McLellan JS. 2020. Cryo-EM structure of the 2019-nCoV spike in the prefusion conformation. Science 367:1260–1263. doi:10.1126/science.abb2507. 32075877 PMC7164637

[10] Walls AC, Park YJ, Tortorici MA, Wall A, McGuire AT, Veesler D. 2020. Structure, function, and antigenicity of the SARS-CoV-2 spike glycoprotein. Cell 181:281–292.e6. doi:10.1016/j.cell.2020.02.058. 32155444 PMC7102599

[11] Hoffmann M, Kleine-Weber H, Pöhlmann S. 2020. A multibasic cleavage site in the spike protein of SARS-CoV-2 is essential for infection of human lung cells. Mol Cell 78:779–784.e5. doi:10.1016/j.molcel.2020.04.022. 32362314 PMC7194065

[12] Jaimes JA, Millet JK, Whittaker GR. 2020. Proteolytic cleavage of the SARS-CoV-2 spike protein and the role of the novel S1/S2 site. iScience 23:101212. doi:10.1016/j.isci.2020.101212. 32512386 PMC7255728

[13] Hoffmann M, Kleine-Weber H, Schroeder S, Krüger N, Herrler T, Erichsen S, Schiergens TS, Herrler G, Wu NH, Nitsche A, Müller MA, Drosten C, Pöhlmann S. 2020. SARS-CoV-2 cell entry depends on ACE2 and TMPRSS2 and is blocked by a clinically proven protease inhibitor. Cell 181:271–280.e8. doi:10.1016/j.cell.2020.02.052. 32142651 PMC7102627

[14] Pallesen J, Wang N, Corbett KS, Wrapp D, Kirchdoerfer RN, Turner HL, Cottrell CA, Becker MM, Wang L, Shi W, Kong WP, Andres EL, Kettenbach AN, Denison MR, Chappell JD, Graham BS, Ward AB, McLellan JS. 2017. Immunogenicity and structures of a rationally designed prefusion MERS-CoV spike antigen. Proc Natl Acad Sci U S A 114:E7348–E7357. doi:10.1073/pnas.1707304114. 28807998 PMC5584442

[15] Rathnasinghe R, Strohmeier S, Amanat F, Gillespie VL, Krammer F, García-Sastre A, Coughlan L, Schotsaert M, Uccellini M. 2020. Comparison of transgenic and adenovirus hACE2 mouse models for SARS-CoV-2 infection. bioRxiv doi:10.1101/2020.07.06.190066. PMC7655046 33073694

[16] ter Meulen J, van den Brink EN, Poon LL, Marissen WE, Leung CS, Cox F, Cheung CY, Bakker AQ, Bogaards JA, van Deventer E, Preiser W, Doerr HW, Chow VT, de Kruif J, Peiris JS, Goudsmit J. 2006. Human monoclonal antibody combination against SARS coronavirus: synergy and coverage of escape mutants. PLoS Med 3:e237. doi:10.1371/journal.pmed.0030237. 16796401 PMC1483912

[17] Yuan M, Wu NC, Zhu X, Lee CD, So RTY, Lv H, Mok CKP, Wilson IA. 2020. A highly conserved cryptic epitope in the receptor-binding domains of SARS-CoV-2 and SARS-CoV. Science 368:630–633. doi:10.1126/science.abb7269. 32245784 PMC7164391

[18] Amanat F, Stadlbauer D, Strohmeier S, Nguyen THO, Chromikova V, McMahon M, Jiang K, Arunkumar GA, Jurczyszak D, Polanco J, Bermudez-Gonzalez M, Kleiner G, Aydillo T, Miorin L, Fierer DS, Lugo LA, Kojic EM, Stoever J, Liu STH, Cunningham-Rundles C, Felgner PL, Moran T, Garcia-Sastre A, Caplivski D, Cheng AC, Kedzierska K, Vapalahti O, Hepojoki JM, Simon V, Krammer F. 2020. A serological assay to detect SARS-CoV-2 seroconversion in humans. Nat Med 26:1033–1036. doi:10.1038/s41591-020-0913-5. 32398876 PMC8183627

[19] Stadlbauer D, Amanat F, Chromikova V, Jiang K, Strohmeier S, Arunkumar GA, Tan J, Bhavsar D, Capuano C, Kirkpatrick E, Meade P, Brito RN, Teo C, McMahon M, Simon V, Krammer F. 2020. SARS-CoV-2 seroconversion in humans: a detailed protocol for a serological assay, antigen production, and test setup. Curr Protoc Microbiol 57:e100. doi:10.1002/cpmc.100. 32302069 PMC7235504

[20] Amanat F, White KM, Miorin L, Strohmeier S, McMahon M, Meade P, Liu WC, Albrecht RA, Simon V, Martinez-Sobrido L, Moran T, Garcia-Sastre A, Krammer F. 2020. An in vitro microneutralization assay for SARS-CoV-2 serology and drug screening. Curr Protoc Microbiol 58:e108. doi:10.1002/cpmc.108. 32585083 PMC7361222

[21] Hassan AO, Case JB, Winkler ES, Thackray LB, Kafai NM, Bailey AL, McCune BT, Fox JM, Chen RE, Alsoussi WB, Turner JS, Schmitz AJ, Lei T, Shrihari S, Keeler SP, Fremont DH, Greco S, McCray PB, Perlman S, Holtzman MJ, Ellebedy AH, Diamond MS. 2020. A SARS-CoV-2 infection model in mice demonstrates protection by neutralizing antibodies. Cell 182:744–753.e4. doi:10.1016/j.cell.2020.06.011. 32553273 PMC7284254

[22] Sun J, Zhuang Z, Zheng J, Li K, Wong RL-Y, Liu D, Huang J, He J, Zhu A, Zhao J, Li X, Xi Y, Chen R, Alshukairi AN, Chen Z, Zhang Z, Chen C, Huang X, Li F, Lai X, Chen D, Wen L, Zhuo J, Zhang Y, Wang Y, Huang S, Dai J, Shi Y, Zheng K, Leidinger MR, Chen J, Li Y, Zhong N, Meyerholz DK, McCray PB, Perlman S, Zhao J. 2020. Generation of a broadly useful model for COVID-19 pathogenesis, vaccination, and treatment. Cell 182:734–743.e5. doi:10.1016/j.cell.2020.06.010. 32643603 PMC7284240

[23] Krammer F. 2020. SARS-CoV-2 vaccines in development. Nature 586:516–527. doi:10.1038/s41586-020-2798-3. 32967006

[24] van Doremalen N, Lambe T, Spencer A, Belij-Rammerstorfer S, Purushotham JN, Port JR, Avanzato VA, Bushmaker T, Flaxman A, Ulaszewska M, Feldmann F, Allen ER, Sharpe H, Schulz J, Holbrook M, Okumura A, Meade-White K, Pérez-Pérez L, Edwards NJ, Wright D, Bissett C, Gilbride C, Williamson BN, Rosenke R, Long D, Ishwarbhai A, Kailath R, Rose L, Morris S, Powers C, Lovaglio J, Hanley PW, Scott D, Saturday G, de Wit E, Gilbert SC, Munster VJ. 2020. ChAdOx1 nCoV-19 vaccine prevents SARS-CoV-2 pneumonia in rhesus macaques. Nature 586:578–582. doi:10.1038/s41586-020-2608-y. 32731258 PMC8436420

[25] Logunov DY, Dolzhikova IV, Zubkova OV, Tukhvatullin AI, Shcheblyakov DV, Dzharullaeva AS, Grousova DM, Erokhova AS, Kovyrshina AV, Botikov AG, Izhaeva FM, Popova O, Ozharovskaya TA, Esmagambetov IB, Favorskaya IA, Zrelkin DI, Voronina DV, Shcherbinin DN, Semikhin AS, Simakova YV, Tokarskaya EA, Lubenets NL, Egorova DA, Shmarov MM, Nikitenko NA, Morozova LF, Smolyarchuk EA, Kryukov EV, Babira VF, Borisevich SV, Naroditsky BS, Gintsburg AL. 2020. Safety and immunogenicity of an rAd26 and rAd5 vector-based heterologous prime-boost COVID-19 vaccine in two formulations: two open, non-randomised phase 1/2 studies from Russia. Lancet 396:887–897. doi:10.1016/S0140-6736(20)31866-3. 32896291 PMC7471804

[26] Zhu FC, Guan XH, Li YH, Huang JY, Jiang T, Hou LH, Li JX, Yang BF, Wang L, Wang WJ, Wu SP, Wang Z, Wu XH, Xu JJ, Zhang Z, Jia SY, Wang BS, Hu Y, Liu JJ, Zhang J, Qian XA, Li Q, Pan HX, Jiang HD, Deng P, Gou JB, Wang XW, Wang XH, Chen W. 2020. Immunogenicity and safety of a recombinant adenovirus type-5-vectored COVID-19 vaccine in healthy adults aged 18 years or older: a randomised, double-blind, placebo-controlled, phase 2 trial. Lancet 396:479–488. doi:10.1016/S0140-6736(20)31605-6. 32702299 PMC7836858

[27] Zhang Y-J, Zeng G, Pan H-X, Li C-G, Kan B, Hu Y-L, Mao H-Y, Xin Q-Q, Chu K, Han W-X, Chen Z, Tang R, Yin W-D, Chen X, Gong X-J, Qin C, Hu Y-S, Liu X-Y, Cui G-L, Jiang C-B, Zhang H-M, Li J-X, Yang M-N, Lian X-J, Song Y, Lu J-X, Wang X-X, Xu M, Gao Q, Zhu F-C. 2020. Immunogenicity and safety of a SARS-CoV-2 inactivated vaccine in healthy adults aged 18–59 years: report of the randomized, double-blind, and placebo-controlled phase 2 clinical trial. medRxiv doi:10.1101/2020.07.31.20161216.

[28] Sun W, McCroskery S, Liu WC, Leist SR, Liu Y, Albrecht RA, Slamanig S, Oliva J, Amanat F, Schaefer A, Dinnon KH, Innis BL, Garcia-Sastre A, Krammer F, Baric RS, Palese P. 2020. A Newcastle disease virus (NDV) expressing membrane-anchored spike as a cost-effective inactivated SARS-CoV-2 vaccine. bioRxiv doi:10.1101/2020.07.30.229120. PMC7766959 33348607

[29] Corbett KS, Edwards D, Leist SR, Abiona OM, Boyoglu-Barnum S, Gillespie RA, Himansu S, Schäfer A, Ziwawo CT, DiPiazza AT, Dinnon KH, Elbashir SM, Shaw CA, Woods A, Fritch EJ, Martinez DR, Bock KW, Minai M, Nagata BM, Hutchinson GB, Bahl K, Garcia-Dominguez D, Ma L, Renzi I, Kong WP, Schmidt SD, Wang L, Zhang Y, Stevens LJ, Phung E, Chang LA, Loomis RJ, Altaras NE, Narayanan E, Metkar M, Presnyak V, Liu C, Louder MK, Shi W, Leung K, Yang ES, West A, Gully KL, Wang N, Wrapp D, Doria-Rose NA, Stewart-Jones G, Bennett H, Nason MC, Ruckwardt TJ. 2020. SARS-CoV-2 mRNA vaccine development enabled by prototype pathogen preparedness. bioRxiv doi:10.1101/2020.06.11.145920. PMC7581537 32756549

[30] Polack FP, Thomas SJ, Kitchin N, Absalon J, Gurtman A, Lockhart S, Perez JL, Pérez Marc G, Moreira ED, Zerbini C, Bailey R, Swanson KA, Roychoudhury S, Koury K, Li P, Kalina WV, Cooper D, Frenck RW, Hammitt LL, Türeci Ö, Nell H, Schaefer A, Ünal S, Tresnan DB, Mather S, Dormitzer PR, Şahin U, Jansen KU, Gruber WC, Group CCT. 2020. Safety and efficacy of the BNT162b2 mRNA Covid-19 vaccine. N Engl J Med 383:2603–2615. doi:10.1056/NEJMoa2034577. 33301246 PMC7745181

[31] Mercado NB, Zahn R, Wegmann F, Loos C, Chandrashekar A, Yu J, Liu J, Peter L, McMahan K, Tostanoski LH, He X, Martinez DR, Rutten L, Bos R, van Manen D, Vellinga J, Custers J, Langedijk JP, Kwaks T, Bakkers MJG, Zuijdgeest D, Huber SKR, Atyeo C, Fischinger S, Burke JS, Feldman J, Hauser BM, Caradonna TM, Bondzie EA, Dagotto G, Gebre MS, Hoffman E, Jacob-Dolan C, Kirilova M, Li Z, Lin Z, Mahrokhian SH, Maxfield LF, Nampanya F, Nityanandam R, Nkolola JP, Patel S, Ventura JD, Verrington K, Wan H, Pessaint L, Ry AV, Blade K, Strasbaugh A, Cabus M, . 2020. Single-shot Ad26 vaccine protects against SARS-CoV-2 in rhesus macaques. Nature 586:583–588. doi:10.1038/s41586-020-2607-z. 32731257 PMC7581548

[32] Keech C, Albert G, Cho I, Robertson A, Reed P, Neal S, Plested JS, Zhu M, Cloney-Clark S, Zhou H, Smith G, Patel N, Frieman MB, Haupt RE, Logue J, McGrath M, Weston S, Piedra PA, Desai C, Callahan K, Lewis M, Price-Abbott P, Formica N, Shinde V, Fries L, Lickliter JD, Griffin P, Wilkinson B, Glenn GM. 2020. Phase 1–2 trial of a SARS-CoV-2 recombinant spike protein nanoparticle vaccine. N Engl J Med 383:2320–2332. doi:10.1056/NEJMoa2026920. 32877576 PMC7494251

[33] Mercier S, Gahéry-Segard H, Monteil M, Lengagne R, Guillet JG, Eloit M, Denesvre C. 2002. Distinct roles of adenovirus vector-transduced dendritic cells, myoblasts, and endothelial cells in mediating an immune response against a transgene product. J Virol 76:2899–2911. doi:10.1128/jvi.76.6.2899-2911.2002. 11861857 PMC136003

[34] Zsengellér Z, Otake K, Hossain S-A, Berclaz P-Y, Trapnell BC. 2000. Internalization of adenovirus by alveolar macrophages initiates early proinflammatory signaling during acute respiratory tract infection. J Virol 74:9655–9667. doi:10.1128/JVI.74.20.9655-9667.2000. 11000238 PMC112398

[35] Coughlan L. 2020. Factors which contribute to the immunogenicity of non-replicating adenoviral vectored vaccines. Front Immunol 11:909. doi:10.3389/fimmu.2020.00909. 32508823 PMC7248264

[36] Zhou Z, Post P, Chubet R, Holtz K, McPherson C, Petric M, Cox M. 2006. A recombinant baculovirus-expressed S glycoprotein vaccine elicits high titers of SARS-associated coronavirus (SARS-CoV) neutralizing antibodies in mice. Vaccine 24:3624–3631. doi:10.1016/j.vaccine.2006.01.059. 16497416 PMC7115485

[37] Li J, Ulitzky L, Silberstein E, Taylor DR, Viscidi R. 2013. Immunogenicity and protection efficacy of monomeric and trimeric recombinant SARS coronavirus spike protein subunit vaccine candidates. Viral Immunol 26:126–132. doi:10.1089/vim.2012.0076. 23573979 PMC3624630

[38] Kam YW, Kien F, Roberts A, Cheung YC, Lamirande EW, Vogel L, Chu SL, Tse J, Guarner J, Zaki SR, Subbarao K, Peiris M, Nal B, Altmeyer R. 2007. Antibodies against trimeric S glycoprotein protect hamsters against SARS-CoV challenge despite their capacity to mediate FcgammaRII-dependent entry into B cells in vitro. Vaccine 25:729–740. doi:10.1016/j.vaccine.2006.08.011. 17049691 PMC7115629

[39] Bisht H, Roberts A, Vogel L, Subbarao K, Moss B. 2005. Neutralizing antibody and protective immunity to SARS coronavirus infection of mice induced by a soluble recombinant polypeptide containing an N-terminal segment of the spike glycoprotein. Virology 334:160–165. doi:10.1016/j.virol.2005.01.042. 15780866 PMC7111832

[40] He Y, Zhou Y, Liu S, Kou Z, Li W, Farzan M, Jiang S. 2004. Receptor-binding domain of SARS-CoV spike protein induces highly potent neutralizing antibodies: implication for developing subunit vaccine. Biochem Biophys Res Commun 324:773–781. doi:10.1016/j.bbrc.2004.09.106. 15474494 PMC7092904

[41] Coughlan L, Vallath S, Saha A, Flak M, McNeish IA, Vassaux G, Marshall JF, Hart IR, Thomas GJ. 2009. In vivo retargeting of adenovirus type 5 to alphavbeta6 integrin results in reduced hepatotoxicity and improved tumor uptake following systemic delivery. J Virol 83:6416–6428. doi:10.1128/JVI.00445-09. 19369326 PMC2698540

[42] Amanat F, Duehr J, Oestereich L, Hastie KM, Ollmann Saphire E, Krammer F. 2018. Antibodies to the glycoprotein GP2 subunit cross-react between Old and New World arenaviruses. mSphere 3:e00189-18. doi:10.1128/mSphere.00189-18. 29720525 PMC5932378

[43] Margine I, Palese P, Krammer F. 2013. Expression of functional recombinant hemagglutinin and neuraminidase proteins from the novel H7N9 influenza virus using the baculovirus expression system. J Vis Exp 2013:e51112. doi:10.3791/51112. PMC3970794 24300384

[44] Amanat F, Duehr J, Huang C, Paessler S, Tan GS, Krammer F. 2019. Monoclonal antibodies with neutralizing activity and Fc-effector functions against the Machupo virus glycoprotein. J Virol 94:e01741-19. doi:10.1128/JVI.01741-19. PMC7022345 31801871

